# Molecular Mechanism of the Anti-Inflammatory Action of Heparin

**DOI:** 10.3390/ijms221910730

**Published:** 2021-10-03

**Authors:** Leandar Litov, Peicho Petkov, Miroslav Rangelov, Nevena Ilieva, Elena Lilkova, Nadezhda Todorova, Elena Krachmarova, Kristina Malinova, Anastas Gospodinov, Rossitsa Hristova, Ivan Ivanov, Genoveva Nacheva

**Affiliations:** 1 Faculty of Physics, Sofia University “St. Kl. Ohridski”, 5, James Bourchier Blvd, 1164 Sofia, Bulgaria; peicho@phys.uni-sofia.bg; 2 Institute of Organic Chemistry with Centre of Phytochemistry, Bulgarian Academy of Sciences, 9, Acad. G. Bonchev Str., 1113 Sofia, Bulgaria; marangelov@gmail.com; 3 Institute of Information and Communication Technologies, Bulgarian Academy of Sciences, 25A, Acad. G. Bonchev Str., 1113 Sofi, Bulgaria; nevena.ilieva@iict.bas.bg (N.I.); elena.lilkova@iict.bas.bg (E.L.); 4 Institute of Biodiversity and Ecosystem Research, Bulgarian Academy of Sciences, 2, Gagarin Street, 1113 Sofia, Bulgaria; nadeshda@abv.bg; 5 Institute of Molecular Biology “Roumen Tsanev”,Bulgarian Academy of Sciences, 21, Acad. G. Bonchev Str., 1113 Sofia, Bulgaria; elenakrachmarova@bio21.bas.bg (E.K.); kristina.malinova94@abv.bg (K.M.); agg@bio21.bas.bg (A.G.); hristova_r@bio21.bas.bg (R.H.); iivanov@bio21.bas.bg (I.I.); genoveva@bio21.bas.bg (G.N.)

**Keywords:** molecular dynamics, molecular modelling, cytokine storm, inflammation, low-molecular-weight heparin (LMWH), IFNγ, IL-6, signalling pathway, COVID-19

## Abstract

Our objective is to reveal the molecular mechanism of the anti-inflammatory action of low-molecular-weight heparin (LMWH) based on its influence on the activity of two key cytokines, IFNγ and IL-6. The mechanism of heparin binding to IFNγ and IL-6 and the resulting inhibition of their activity were studied by means of extensive molecular-dynamics simulations. The effect of LMWH on IFNγ signalling inside stimulated WISH cells was investigated by measuring its antiproliferative activity and the translocation of phosphorylated STAT1 in the nucleus. We found that LMWH binds with high affinity to IFNγ and is able to fully inhibit the interaction with its cellular receptor. It also influences the biological activity of IL-6 by binding to either IL-6 or IL-6/IL-6Rα, thus preventing the formation of the IL-6/IL-6Rα/gp130 signalling complex. These findings shed light on the molecular mechanism of the anti-inflammatory action of LMWH and underpin its ability to influence favourably conditions characterised by overexpression of these two cytokines. Such conditions are not only associated with autoimmune diseases, but also with inflammatory processes, in particular with COVID-19. Our results put forward heparin as a promising means for the prevention and suppression of severe CRS and encourage further investigations on its applicability as an anti-inflammatory agent.

## 1. Introduction

Heparin is a member of the glycosaminoglycan (GAG) family, a linear highly sulfated polysaccharide chain of variable length, composed of repeating disaccharide units. This structure defines one of the most prominent characteristics of the molecule—its high-density negative electric charge. This is the main reason for the high biological activity of heparin. It binds to more than 700 proteins. Heparin is widely used in medical practice as an anticoagulant, with generally mild side effects. The molecular weight of natural heparin (also known as unfractionated heparin, UFH) varies between 5 kDa and more than 40 kDa, depending on the chain length. Its fractionated form, the low-molecular-weight heparin (LMWH), in which at least 60% of the chains are below 8 kDa of weight, is often preferred in clinical practice because of its more predictable pharmacokinetics and milder side effects [1].

Despite its known anticoagulant activity, the biological function of heparin remains unclear. Heparin alters cytokine levels [2] and its derivatives were shown to be capable of acting at several points in the inflammatory process [3]. Thus, one of its biological roles may be to dampen the effects of the sudden release of large amounts of cytokines following infection or injury (see [4] and references therein).

The severe phase of COVID-19 is characterised by a high level of expression of interferon-stimulated genes with proinflammatory activities, showing the immunopathological role of IFNγ response [5]. Among the elevated cytokines, IFNγ and the IFNγ-stimulated chemokines are especially prominent already in the very early fever days. Downregulation of the activity of two key cytokines, IFNγ and IL-6, appears as a promising strategy towards the reduction or even reversal of the development of the cytokine storm.

It is known that IFNγ binds with high affinity to glycosaminoglycans (GAGs), in particular heparan sulfate (HS) and heparin [6,7,8]. These interactions affect the activity, physico-chemical properties, and proteolytic processing of the C-terminal domain of the cytokine. IL-6 also interacts with heparin, forming a stable complex [9]. However, thus far, the use of heparin as an effective IFNγ or IL-6 inhibitor directly preventing the formation of biologically active complexes (IFNγ/ IFNγRα, resp. IL-6/IL-6Rα/gp130) was given little to no attention. 

The objective of our investigations was to shed light on the molecular mechanism of the anti-inflammatory action of heparin and argue its potential for the prevention and suppression of the development of the cytokine storm in conditions characterised by overexpression of certain cytokines. In particular, we investigated its ability to bind to IFNγ and IL-6 cytokines and inhibit their signalling pathways that allows for modulation of the development of a cytokine storm, including in patients with acute COVID-19.

## 2. Results

### 2.1. Simulation of Heparin Hexasaccharides’ Interaction with IFNγ

Interferon-gamma (IFNγ) is a pleiotropic cytokine. The mature form of human IFNγ is a non-covalent homodimer, each of the monomers consisting of 143 amino acids (aa), 62% of which are arranged in 6 α-helices (A to F), associated with short unstructured regions, and a highly flexible C-terminal domain (21 aa). The 3D structure of IFNγ in complex with its receptor—two non-covalently associated chains, IFNGR1 and IFNGR2—is resolved and the binding sites are localised on the IFNγ molecule in the loop between helices A and B (residues 18–26), His^111^ and a short putative area (residues 128–131) in the flexible C-terminal domain [10,11]. IFNγ is a basic cytokine, with the full-length homodimer having a net charge of +18e. The positive charge is concentrated mainly in the domains D1 (^125^LysThrGlyLysArgLysArg^131^, charge +5e) and D2 (^137^ArgGlyArgArg^140^, charge +3e) of the C-termini. In addition, there is also the short basic domain ^86^LysLysLysArg^89^ in the lower part of the cytokine globule. LMWH sugars are highly negatively charged, each chain of the selected representative LMWH structure having a net charge of –9e. Therefore, the interaction between IFNγ and the hexasaccharides must be dominated by a very intensive electrostatic attraction.

**Figure 1:**
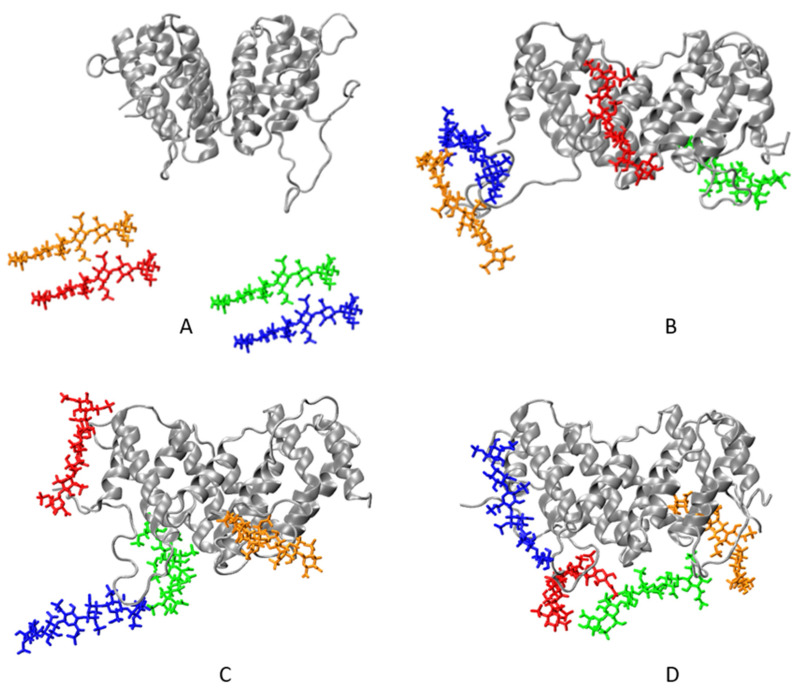
Initial (**A**) and final (**B**–**D**) configurations of the three IFNγ–LMWH hexasaccharide binding simulations. The protein is shown in gray ribbon, and the hexasaccharide chains are coloured as follows: dp6_1—green, dp6_2 –orange, dp6_3—blue, and dp6_4—red.

**Figure 2:**
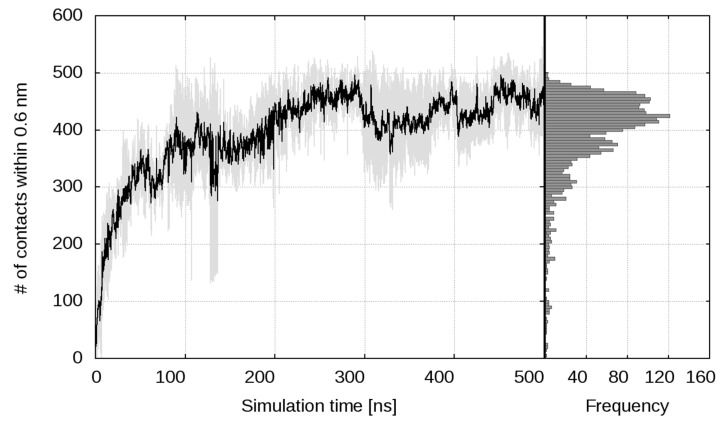
Pair contacts between IFNγ and the hexasaccharides. Number of contacts (averaged over the three independent simulations) as a function of the simulation time between any pair of atoms of IFNγ and any of the four hexasaccharides within 0.6 nm, with the standard deviation band (in grey) and number-of-contact frequencies.

Three MD simulations of 500 ns each, starting from the same initial configuration, but with different initial velocities (Figure 1), confirmed these expectations. The details slightly vary from one simulation to another, but the general pattern remains the same. The first carbohydrate attaches to the cytokine in just 1–2 ns. Another nanosecond later, three of the four saccharides have formed at least one contact with the cytokine. Within the next 35–40 ns, the carbohydrate chains move closer and closer to the protein molecule and form more and more contacts, until all three sugars lie down on the protein surface and the number of contacts curve plateaus. The number of contacts between the protein and the sugars as a function of simulation time is shown in Figure 2, averaged over the three runs. Individual curves are shown in Figure S1 in the Supplementary Materials. Complex formation dynamics is also reflected by the solvent accessible surface area of the carbohydrates, as shown in Figure S2 in the Supplementary Materials. With three hexasaccharides attached to the cytokine, the complex already has a negative net charge. Thus, the fourth LMWH chain binds to IFNγ with some lag, between 80th and 200th nanosecond in the different runs, finding a local (and already exposed) positive-charge surface concentration, which saturates the number of contacts.

LMWH binding has little to no influence on the IFNγ globule, as seen in the secondary structure plots of the apo-form simulation and the three independent hexasaccharide-binding simulations (Figure S3a,b in the Supplementary Materials). At the same time, an impairment of the cytokine’s binding affinity to its receptors is to be expected, already on the basis of a noticeable decrease of the solvent accessible surface area of the C-termini of the cytokine (Figure S4 in the Supplementary Materials). Upon binding of the carbohydrates, the SASA of the C-termini, averaged over the last 100 ns of the simulations, decreases by more than 25%—from 49.8 ± 2.3 nm^2^ to 37 ± 2.6 nm^2^.

**Figure 3:**
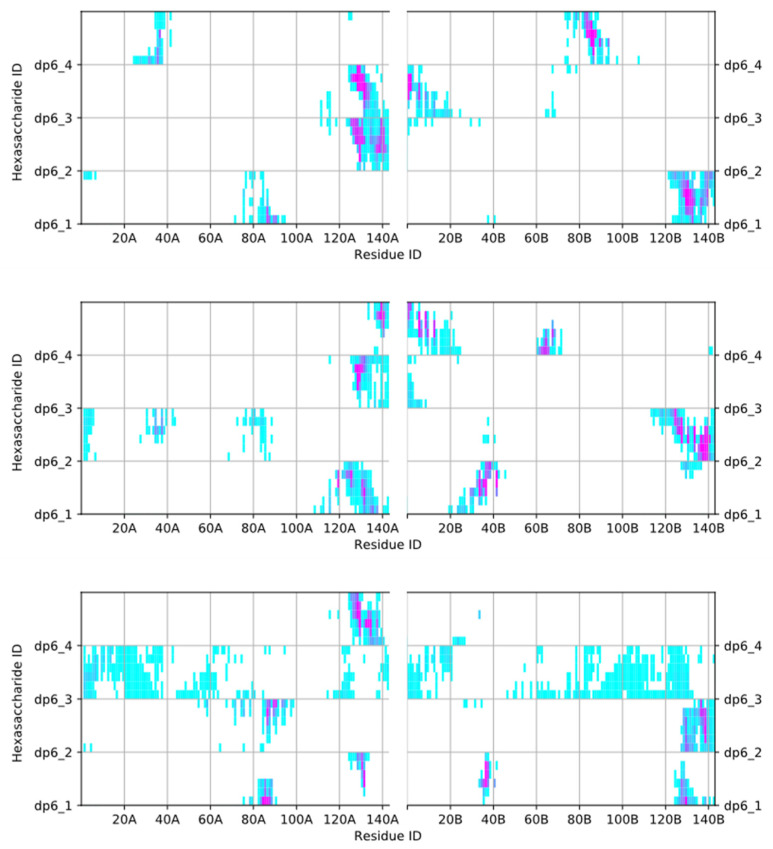
Contact map of the IFNγ/hexasaccharide complex. Contacts within 0.6 nm formed between each of the four hexasaccharides and the two monomers of IFNγ. Contact occupancy within the last 250 ns of the three simulations (upper, middle, and lower panels, resp.) ranges from 0 (cyan) to 1 (purple).

In view of the high negative-charge density of the LMWH molecules, it is to be expected, that the latter will interact with the positively charged parts of the IFNγ molecule (see above). Figure 3 shows a contact map of the close interactions of the four hexasaccharides and the two IFNγ monomers. Indeed, the sugars bind to exactly these three domains. The carbohydrates form the most contacts with the flexible C-termini of the cytokine (Leu^120^-Gln^143^), in particular with their two D1 and D2 basic domains. The LMWH chains also bind favourably to the solvent-exposed parts of helices D and E in the globule (Lys^74^-Phe^92^), which include the highly positively charged tetrapeptide ^86^LysLysLysArg^89^. Another interaction site for the sugars are the positively charged N-terminal NH_3_ group of Gln^1^, Asp^2^, Lys^6^, and Lys^12^-Lys^13^, as well as the sequence ^34^LysAsnTrpLys^38^ at the end of helix B. The IFNγ/LMWH saccharide complexes are very stable. The most stable complexes are formed between the sugars and the D1 and D2 domains and also the ^86^LysLysLysArg^89^ tetrapeptide. In general, once a carbohydrate chain attaches to one of these highly positively charged domains, it does not dissociate from the protein. These complexes are stabilised through a fairly high number of hydrogen bonds. In the last 250 ns of the simulation, the complexes are characterised by between 7 and 14 hydrogen bonds. The interaction between the sugars and the N-terminal part of the cytokine (i.e., aa 1–20, and 34–38) is less intense, hence these complexes are not as stable and are characterised by a lower number of hydrogen bonds—between 2 and 4 on average in the second halves of the simulations. The hydrogen bonds between each hexasaccharide and the IFNγ homodimer are shown in Figure S5 in the Supplementary Materials. 

IFNγ finds its receptor via electrostatic attraction and when its net positive charge is neutralised or even effectively made negative, this is no longer possible. The electrostatic potential surface of the input protein configuration, showing the surface distribution of charges in the apo form, and in the IFNγ/LMWH complex is depicted in Figure S6 in the Supplementary Materials. The IFNγ/IFNγR1 complex formation requires almost perfect collinearity of the dipole moments of the two molecules. With the substantial deformation of the potential surface after LMWH recruitment, this is no longer possible.

Our simulations show that LMWH binds to the C-termini of IFNγ with high affinity, forming very stable complexes due to the strong electrostatic attraction. After binding of two or more LMWH hexasaccharides, the complex changes its net charge from positive to negative, but continues to recruit more carbohydrates. However, this additional binding of LMWH chains is possible only if the carbohydrates are in relatively close proximity to the cytokine, so that they are able to experience the local effects of the varying surface charge density of the globule. At larger distances (i.e., low concentrations of heparin in the circulation) the LMWH sugars will only “feel” the presence/absence of a net positive charge of the protein, and once enough carbohydrates bind to it and neutralize it, no further binding will take place. The net-charge change due to LMWH binding impedes further interaction of the cytokine with the extracellular part of the IFNγR1 (also negatively charged), which is the first necessary step in the IFNγ transduction pathway. The in silico simulation data explains the obtained experimental results. These findings put forward heparin as an effective inhibitor of IFNγ and, as such, a good candidate for influencing the development of the cytokine storm. As discussed above, already two heparin molecules of the type taken as an example are enough to make IFNγ “blind” and in that sense, the particular structure of the LMWH chosen is of no importance.

### 2.2. Effect of Heparin on the Activity of IFNγ

Upon binding to its receptor, IFN**γ** activates the JAK/STAT1α signal transduction pathway. STAT1 initiates the transcription of target genes upon translocation into the nucleus following phosphorylation and a subsequent dimerisation. Here, the effect of LMWH was studied through the antiproliferative activity of IFNγ. It was measured by a modified kynurenine bioassay [12] run with a referent recombinant IFNγ having specific biological activity of 10^6^ IU/mg [13]. Preformed monolayers of WISH cells were pre-incubated with various concentrations of LMWH and then treated with 100 ng/mL (100 IU/mL) IFNγ. The antiproliferative activity of IFNγ in this assay is due to the induction of indoleamine-2,3-dioxygenase (IDO), which is the first and rate limiting enzyme in the tryptophan catabolism, catalysing oxidative cleavage of tryptophan to *N*-formylkynurenine.

**Figure 4:**
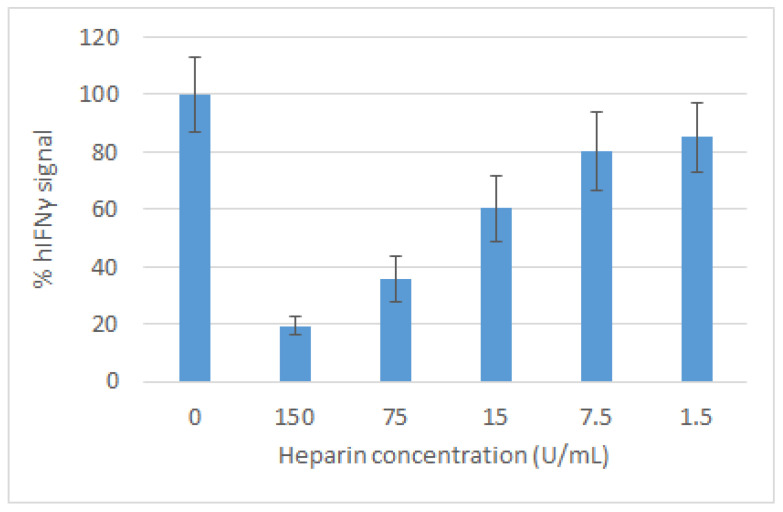
Inhibitory effect of LMWH on the production of IDO induced by 100 IU/mL recombinant IFNγ. After subtraction of the blank value, the absorbance obtained from cells treated with a mixture of IFNγ and LMWH is related to that obtained from cells treated with IFNγ only, taken as 100%. The bars represent the statistical errors.

The hydrolysis of the latter transforms it into kynurenine. Its interaction with Ehrlich’s reagent leads to the formation of a compound, absorbing light with wave-length of 490 nm. As seen in Figure 4, LMWH inhibited the antiproliferative activity of IFNγ in a concentration-dependent manner with concentration of about 35 IU/mL reducing it to 50% of the control level. The addition of LMWH at 150 IU/mL was not toxic to the cells, but abolished the induction of IDO by up to 80%.

**Figure 5:**
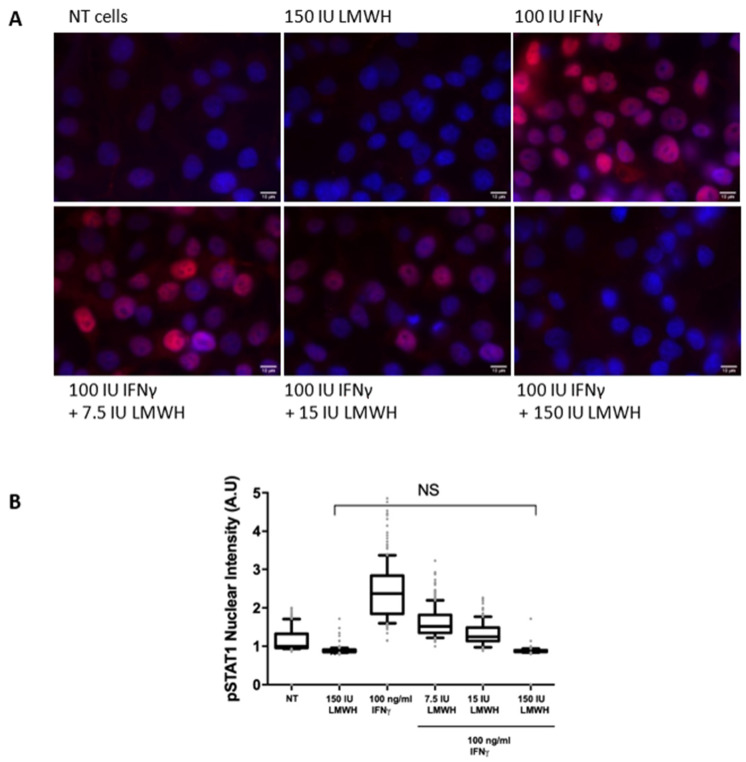
Translocation of the phosphorylated STAT1 into the cell nucleus. Translocation of the phosphorylated STAT1 into the cell nucleus. (**A**) WISH cells were treated with LMWH or IFNγ only, or with IFNγ after pretreatment with LMWH at the indicated concentrations, and fixed and stained with an antibody against pSTAT1 (Tyr701). The nuclei were counterstained with DAPI. Representative images are shown; scale bar, 10 µm; (**B**) Distribution of nuclear intensity of pSTAT1 (Tyr701) of individual nuclei from (A). Data are from 3 independent experiments (*n* > 200 per condition per experiment); NS—non-significant (two-tailed unpaired Student’s *t*-test).

In order to further investigate the effect of LMWH on IFNγ signalling inside the stimulated cells, we studied the translocation of phosphorylated STAT1 in the nucleus using specific antibodies. The endogenous phosphor-STAT1 (Tyr^701^) was visualised by immuno-fluorescence staining of fixed WISH cells after 15 min induction with 100 ng/mL (100 IU/mL) IFNγ alone or in the presence of various concentrations of LMWH (Figure 5). At heparin concentration of 150 U/mL, we observed a full inhibition of the phosphorylation of STAT1 and, therefore, its translocation into the nucleus, meaning a suppression of the IFNγ signalling. 

The obtained results indisputably show that LMWH abolishes the biological activity of IFNγ. In order to shed light on the mechanism of action of LMWH, we performed MD simulation of the interaction of heparin hexasaccharides with the IFNγ molecule.

### 2.3. LMWH Binding to the IL-6 and IL-6/IL-6Rα Complex 

Interleukin 6 (IL-6) is a member of the IL-6 cytokine family, secreted by many cell types upon stimulation during infection, inflammation, or cancer [14]. IL-6 plays an important role in the regulation of the responses of B- and T-lymphocytes and coordination of innate and adaptive immune systems. IL-6 is a 26 kDa, 212-residue bundle cytokine shaped in four α-helical parts with two extensive loop regions situated between helices A and B and helices C and D [15]. It binds to the IL-6 receptor (IL-6Rα), an 80 kDa protein devoid of signalling capacity. Subsequently, a triple complex is formed upon binding to another membrane protein, glycoprotein 130 (gp130, also known as IL-6Rß and acting as a signalling receptor for the whole IL-6 family) which dimerises and initiates intracellular signalling [16]. The gp130 receptor is ubiquitously expressed on the cell surface, whereas IL-6Rα is found only on a few cell types, such as hepatocytes, some leukocytes, and epithelial cells [17]. IL-6 exhibits affinity only to IL-6Rα, not to gp130. The complex IL-6/IL-6Rα/gp130 homodimerises to form a hexamer that is a necessary condition for the initiation of the IL-6 intracellular signalling. As a prerequisite for the complex-formation studies, all binding sites in the triple complex IL-6/IL-6Rα/gp130 were scrutinised. The complex structure prepared as described in Section 4.6 is shown in Figure S7 in the Supplementary Materials. Three binding sites were identified in IL-6 (for details see [18]; we keep to the numbering convention in [9]: site I, comprising residues Arg^30^, Leu^33^, Lys^54^ (helix A) and Leu^178^, Arg^179^, Arg^182^, Gln^183^ (helix D), involved in the interaction of IL-6 with IL-6Rα receptor; site II, comprising residues Arg^24^, Gln^28^, Tyr^31^, Asp^34^ (helix A) and Glu^110^, Met^117^, Val^121^, Gln^124^, Phe^125^ (helix C), involved in the binding of IL-6 to gp130 receptor; site III, involved in the formation of the hexamer complex.

**Figure 6:**
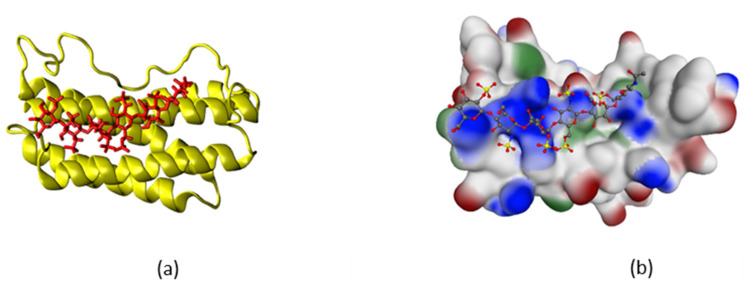
The IL-6/LMWH complex. (**a**) IL-6 in yellow, heparin in red; (**b**) IL-6 molecule presented with its SAS (hydrophobic regions in green, positively charged regions in blue, and negatively charged regions in red), heparin coloured by atom type.

It is noteworthy that both IL-6 and IL-6/IL6Ra complex are electrostatically neutral molecular systems, thus no intense Coulomb attraction, similar to the one observed in the IFNγ–LMWH simulation, was to be expected. Since we focussed on the anti-inflammatory action of heparin and not on scrutinizing the binding itself, the carbohydrate was placed in the proximity of the binding sites of interest, and then we studied the stability of the so-formed complexes by means of MD. A 250-ns simulation of IL-6/LMWH complex was performed with the initial conformation chosen with account for the identified binding sites, specifically targeting the positively charged amino acid residues of IL-6, involved in purely polar interactions between IL-6 and IL-6Rα (Figure 6a). Comparison of the secondary-structure plots of the apo-form 150-ns reference simulation and the 250-ns simulation of the IL-6/LMWH complex revealed no substantial structural changes in IL-6 induced through the LMWH binding, but only a minor deformation of the α-helical structure up to Arg^24^ and a formation of some turns in the region Met^49^-Ser^52^ (Figure S8 of the Supplementary Materials). The latter, however, cannot be attributed to LMWH influence, as the same happened in a simulation of the IL-6/IL-6Rα complex and the heparin in this case was positioned away from this site (Figure S9 of the Supplementary Materials).

In the IL-6/LMWH simulation, a stable conformation was achieved, with heparin binding to IL-6 at Arg^24^, Arg^30^, and Arg^40^ from helix A and Lys^171^, Arg^179^, and Arg^182^ from helix D. All these interactions are electrostatic in nature, determined by the strong attraction between the positively charged residues of IL-6 and the negatively charged heparin (Figure 6b). Close contacts (defined as the distance between any atom in the heparin molecule and any atom of the given residue being below 3 Å) are formed also with Lys^27^, Leu^33^, Ser^37^, Cys^50^, Glu^51^, and Gln^175^. Thus, in this interaction, four out of seven residues from site I, among them the key residues Arg^30^, Arg^179^, and Arg^182^, are engaged, as well as Arg^24^ and the immediate neighbor of Gln^28^ from site II. 

**Figure 7:**
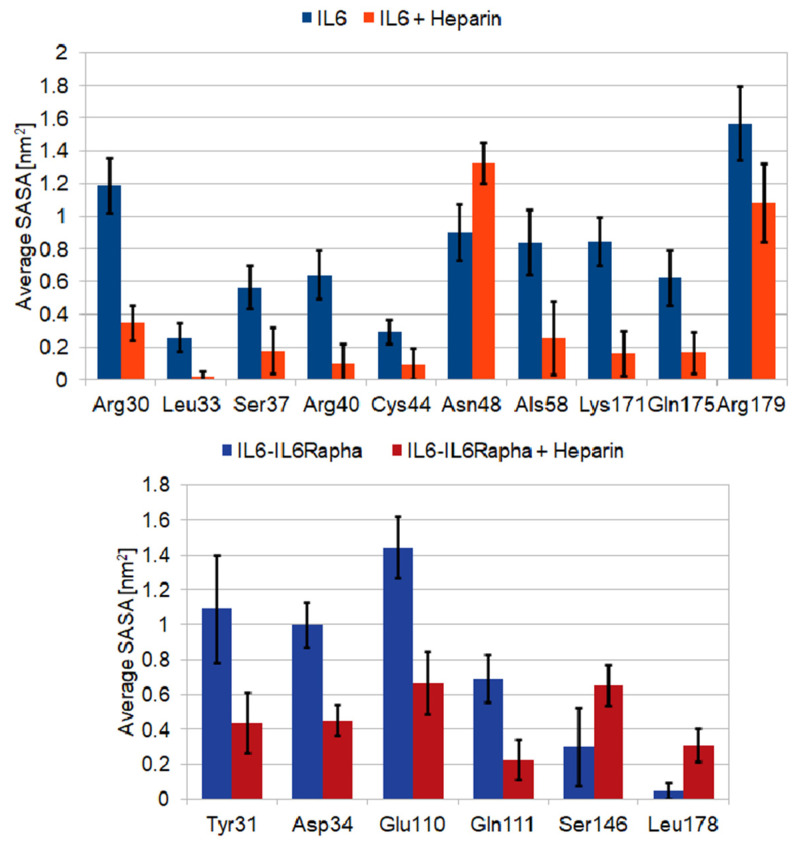
Average SASA values for the most affected through LMWH binding residues (SASA change exceeds the standard deviation). IL6 (**top** panel); IL-6/IL-6Rα complex (**bottom** panel).

Upon heparin binding, the SAS area of the solvent-exposed residues, in particular those involved in the triple-complex formation, may change. To estimate possible critical changes, we analysed the contribution of each residue to the solvent accessible surface area over the whole trajectory for both simulations. In Figure 7 (top panel), these values for the residues for which the changes exceed the standard deviation are shown. Among the latter are all key residues of site I discussed above.

Site I accounts for IL-6 binding to the IL-6Rα receptor, thus the heparin molecule impairs this interaction, thereby inhibiting the formation of the IL-6/IL-6Rα complex. The involvement of the only charged residue from site II, Arg^24^, is an indication for a possible influence on the IL-6 binding to the second receptor, gp130, as well.

**Figure 8:**
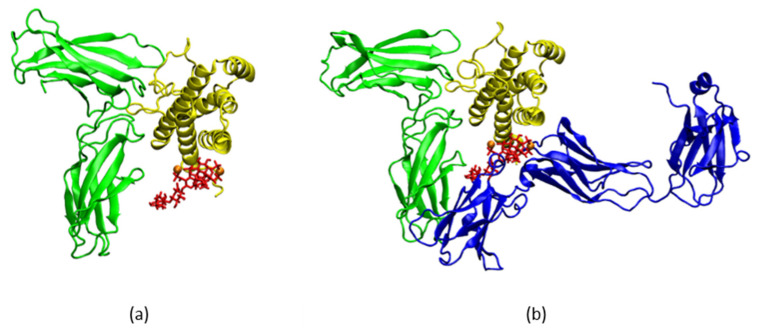
The IL-6/IL-6Rα/LMWH/2Mg^2+^ complex. (**a**) IL-6 in yellow, heparin in red, IL-6Rα in green; Mg^2+^ ions presented as orange spheres; (**b**) The same complex, with gp130 receptor (in blue) superimposed.

In studying the possibility for heparin to hinder through its binding to the IL-6/IL-6Rα complex, the subsequent formation of the biologically active triple complex with gp130, one putative position was identified, in which heparin binds to Arg^30^ of IL-6 helix A and Lys^252^ of IL-6Rα and comes in close proximity to Tyr^31^ of IL-6 helix A. This position is of particular interest because, based on mutation studies [19], Tyr^31^ is considered to be of key importance for the biological activity of IL-6. Clear stability was observed when two Mg^2+^ ions were added in a total of 500 ns simulation. The final conformation of this simulation is depicted in Figure 8a. This result is not surprising, taking into consideration the findings about the Ca^2+^-dependent action of heparin outside the anticoagulation context (see, e.g., [20]).

Comparison of the secondary-structure plots from this binding simulation and the 150-ns reference simulation of the IL-6/IL-6Rα complex reveals even lesser structural changes than in the case of IL-6 alone—the only worth mentioning being the turns stabilization in the region Lys^252^-Gln^255^ of IL-6Rα (Figure S9 in the Supplementary Materials). Residues Leu^19^, Arg^30^, Tyr^31^, Asp^34^, Glu^110^, and Gln^111^, the last four being part of IL-6 binding site II, form close contacts with the heparin molecule, all of them experiencing a substantial decrease in the average SASA value in the bound state (Figure 7, bottom panel).

With the gp130 receptor superimposed onto the simulated system (Figure 8b), the significant impact potential of LMWH on the formation of the IL-6/IL-6Rα/gp130 complex becomes apparent. Not only does heparin bind to Arg^30^ and come in close proximity to the key residue Tyr^31^, but it is also positioned in front of helices A and C of IL-6, thus covering the Tyr^31^-part of IL-6 binding site II and preventing helix C from getting close enough to gp130 to form a complex. The involvement of LMWH impairs IL-6 and its two receptor parts in forming a biologically active complex, meaning an interruption of the IL-6 signalling pathway.

## 3. Discussion

IFNγ can induce both pro- and anti-inflammatory responses. This allows it to tailor the immune response either for defense against infection or towards maintaining the homeostasis of the host [21]. When overexpressed, IFNγ is a key factor in the pathogenesis of autoimmune diseases, uncontrolled inflammation, atherosclerosis, neurodegenerative diseases, and cancer [21,22,23]. Thus, inhibition of IFNγ signal transduction may be useful not only in the case of CRS, but also in the treatment of such diseases.

Our experimental and computational investigations show that LMWH binds with high affinity to IFNγ and, at a certain concentration, is able to fully inhibit the interaction with its cellular receptor, thus blocking the IFNγ signalling pathway and the expression of the IFNγ induced proteins. This observation not only reveals heparin as a potent candidate for the regulation of the IFNγ overexpression, but in a more general perspective, explains the molecular mechanism of its anti-inflammatory action. In particular, it can be helpful in the therapy of autoimmune diseases, acute inflammatory processes, and cytokine release syndrome.

Under normal conditions, the levels of IL-6 are very low, but they raise many thousand-fold in inflammatory states. This is the reason for IL-6 being used as a biomarker for the inflammation levels in patients with cancer, infections, autoimmune diseases, or COVID-19 [24,25]. Inhibition of the IL-6/gp130/STAT3 pathway is considered to have therapeutic potential in such conditions [9,26]. At present, an antibody that blocks site I, siltuximab [27,28], has been approved as a drug. Neutralizing antibodies that block IL-6 receptors outside of the cell or small chemicals that target the involved kinases and transcription factors within the cell have been developed, some of which are approved as biologics (e.g., tocilizumab [29]) and others are in trials. However, the major concern regarding their use is related to the side effects, increased risk of bacterial and viral infections, and their lack of specificity [30].

Our simulations show that a feasible alternative for blocking the binding of IL-6 to its receptors is the application of LMWH. The heparin molecule, forming a stable complex with the cytokine, blocks its binding site I and prevents helices A and D from getting in close contact with IL-6Rα. Thus, similar to the case of IFNγ, heparin breaks the cytokine signalling pathway by inhibiting the formation of the IL-6/IL-6Rα complex. In addition, binding to the complex IL-6/IL-6Rα, heparin blocks binding site II of IL-6, thus preventing helices A and C from being in a proper position to bind to the gp130 receptor. Our computational results explain the experimental observations on the heparin-binding properties of IL-6 [9] and allow us to conclude that LMWH is a strong candidate for IL-6 activity inhibitor, especially in the context of the trans-signalling mechanism. 

GAGs are universally present on cell surfaces and might be expected to serve as broad-spectrum and relatively non-specific receptors for virus binding [31]. Heparin as a potential inhibitor of viral attachment has been demonstrated for DENV [32]. In recent studies [33,34,35], viral attachment to GAGs was reported for IFNα/βBP from Japanese encephalitis virus, variola, monkeypox viruses, and hepatitis B. The effectiveness of heparin and its derivatives has been reported in preventing cell infection by the influenza virus H5N1 [36], as well as in ZIKV-induced cell death [37]. Binding to the SARS-CoV-2 spike-protein in the receptor-binding domain, heparin inhibits virus invasion by 70% due to the induced conformational changes there [38]. 

GAGs and heparin, in particular, have been suggested as playing a protective role in the inflammation response [39], which has been interpreted as involving (among other things) the inhibition of elastase and the interaction with several cytokines [3,40]. Thus, a number of heparin applications may be rooted in its ability to moderate inflammation (see [4,41]).

The molecular mechanism of the anti-inflammatory action of LMWH does not depend on the virus type and, in general, the cause of the acute inflammatory process. This broadens its application range even more—for the treatment of viral infections leading to acute inflammatory conditions characterised by increased cytokine levels, in particular those of IL-6 and IFNγ.

Overexpression of IFNγ is also observed in a number of autoimmune diseases. A possible treatment strategy consists in inhibiting the biological activity of IFNγ. The results of our study reveal the capability of heparin to influence the development of such autoimmune diseases.

The results presented here expand the scope of heparin, providing a solid background for an expected significant improvement of the therapy outcome, whereby the action of heparin will be threefold—anticoagulant, anti-inflammatory, and antiviral.

An added benefit is that heparin is a well-known and widely used medication and its side effects have been studied in detail (see [42,43] and the references therein). This greatly facilitates the expansion of its scope; the general limitations for LMWH use remain valid in these cases as well.

### The COVID-19 Case

COVID-19, the disease caused by the new coronavirus SARS-CoV-2 [44], accounts so far for almost 220 million cases worldwide and over 4.5 million deaths (as of 31 August 2021). The majority of COVID-19 patients have no or only mild symptoms. Severe symptoms like fever and pneumonia, escalating to acute respiratory distress syndrome, have been reported in up to 20% of the clinical cases, most of them accompanied by a cytokine release syndrome (CRS) [45]. The cytokine storm is a state of overreaction of the immune system, characterised by an uncontrolled overproduction of pro-inflammatory cytokines, with interferons and interleukins being among the main components involved in its development [46,47]. 

So far, there are no specific drugs approved as an effective means for fighting COVID-19. Timely control of the cytokine storm in its early stage is the key to improving the treatment success rate and reducing the mortality rate in patients with COVID-19. Recently, a number of specific anti-cytokine approaches have been proposed for the treatment of the CRS, based on drugs targeting IL-1, IL-6, IL-18, and IFNγ [48]. Thus, the inhibition of the activity of the two key cytokines, IFNγ and IL-6, appears as a promising strategy towards the reduction or even reversal of the development of the cytokine storm. Low-molecular-weight heparin (LMWH), being able to bind to both these cytokines, is a possible candidate for the realisation of such a strategy.

In the case of COVID-19, heparin is used as an anticoagulant following the observation that patients in the acute stage develop diffuse alveolar damage with extensive pulmonary coagulation activation resulting in the formation of hyaline membranes in the air sacs and fibrin deposition [49]. A wide international trial on the treatment of COVID-19 patients with nebulised heparin was initiated [50]. Reduced mortality in severe patients treated with heparin was reported in [51]. A similar response—reduced severity and mortality—was observed also in hemodialysis patients who were treated with heparin or other anticoagulants according to the standard protocols [52].

The dynamics of lymphocyte subsets and cytokine profiles and their correlations in patients with COVID-19 were studied in [53]. In a retrospective clinical study [54], it was reported that LMWH not only improves the coagulation dysfunction of COVID-19 patients, but also significantly reduces the levels of the inflammatory cytokines and virions, thus promoting the restoration of lymphocyte levels and faster recovery. 

These multiple clinical observations strongly support the conclusion based on our own investigations, which put forward heparin as a promising means for the prevention and suppression of the development of severe CRS in acute COVID-19 patients and encourage further investigations on its applicability as an anti-inflammatory agent. The decision to start the application of LMWH in COVID-19 patients and its dosage go beyond the scope of our study and should be further determined by clinicians, based on a careful evaluation of certain blood parameters. As indication, the observations from clinical studies that showed a significant increase in the levels of IL-2, IL-6, IL-10, and hINFγ at the beginning of the acute phase of the disease, together with an overall decline of lymphocytes including CD4+ and CD8+ T-cells and B-cells, and NK cells [55] might be helpful for an orientation towards a treatment begin with or shortly before the onset of the acute phase. The doses also need to be elaborated, but our results suggest an orientation towards the upper limit of the dosage scale.

## 4. Materials and Methods

### 4.1. Explicit Solvent MD Simulations

The MD simulations in this study were performed with the GROMACS software package [56,57,58]. Unless specified otherwise, the MD protocol consisted of a force field parameterization, structure-in-vacuum energy minimization, solvation, charge neutralization through Na^+^ and Cl^-^ ions at 0.15 M concentration, energy minimization, MD run with position restraints on heavy solute atoms (PR run), and production MD runs in the NPT ensemble. The temperature was kept constant by applying Berendsen [59] and V-rescale [60] thermostats during PR and production runs, respectively. Berendsen barostat was used in equilibration MD runs and Parrinello–Rahman barostat—for the production MD runs [61]. Long-range electrostatic interactions were treated by applying the Particle Mesh Ewald algorithm [62] with a 1.2 nm cut-off radius for short-range interactions. Switch function with a cut-off radius of 1.2 nm and a switching distance of 1 nm was used to calculate the Van der Waals interactions. We used the leap-frog integration algorithm with 2 fs time step, the covalent bonds connecting hydrogen atoms with the solute heavy atoms were kept with fixed length by the LINCS algorithm [63] and the RATTLE algorithm [64] was used to keep the solvent molecules rigid.

### 4.2. Heparin Hexasaccharide Structure

Heparin is composed of repeating disaccharide units of 1,4 linked α-l-iduronic or β-d-glucuronic acid (d-GlcA), and α-d-glucosamine (d-GlcN). The predominant substitution pattern comprises 2-*O*-sulfation of the iduronate residues and *N*- and 6-*O*-sulfation of the glucosamine residues [4,65]. Based on literature data [20,66,67], a preference was given to α-l-IdoA(2S) (1→4) β-d-GlcNS (1→4) α-l-IdoA(2S) (1→4) β-d-GlcNS(6S) (1→4) β-d-GlcA(1→4) β-d-GlcNAc(6s) (shown in Figure S10 in the Supplementary Materials) to be used as a model sequence for a general LMWH molecule. The chosen oligosaccharide chain has a net charge of –9e. The Glycan Reader and Modeler module [68] of the CHARMM-GUI server [69] was used for the generation of a three-dimensional structure, corresponding to the chosen carbohydrate sequence, as well as a topology using the latest version of the CHARMM36 carbohydrate force field [70]. The topology was converted to a GROMACS-compatible topology using the parmed module of Ambertools 16 [71].

### 4.3. Human Interferon Gamma

The 3D model of reconstructed and folded full-length IFNγ was used as the initial structure for the IFNγ-associated simulations. To obtain it, the missing in the crystallographic structure (PDB ID 1fg9 [11]) C-termini were reconstructed in a completely extended backbone conformation. The obtained from a 200-ns MD folding simulation trajectory was clustered [72]. Starting from the structure of the centroid of the largest identified cluster, three independent 500-ns folding simulations with different random generator seeds were performed [73]. The three trajectories were subjected to a fitting procedure and then clustered using the independent parameter ρ [74]. The largest cluster centroid was selected as an input structure. 

### 4.4. Human Interleukin 6

The crystal structure of human interleukin-6 with PDB ID 1ALU [75] was taken from [76]. The missing residues, ^52^SerSerLysGluAlaLeuAlaGluAsn^60^, were added using the loop-modeling interface [77] to MODELLER [78] of UCSF Chimera [79]. The initial structure of the IL-6 molecule for all subsequent IL-6 related studies was prepared with a 10-ns production MD run.

### 4.5. IL-6/IL-6Rα/gp130 Complex

The crystal structure of human IL-6/IL-6Rα/gp130 complex with PDB ID 1P9M [80] was taken from [76]. The 3D structure for interaction analysis was prepared by a 10-ns MD simulation as described above.

### 4.6. Heparin–Protein Complexes Simulations

For studying the interaction of IFNγ with LMWH, four sample LMWH chains were placed near the two C-termini of the homodimer, with a minimal distance between the carbohydrates and the protein of 1.6 nm. The MOE software package [81] was employed to prepare the initial structures of the complexes of IL-6 and heparin, and IL-6/IL6Rα and heparin. For the heparin molecules, initial positions in close proximity to IL-6 binding sites I and II, as indicated in [80], were chosen. For structure visualisation, the VMD software was used [82].

### 4.7. Reference Simulations

For more accurate addressing of cytokines’ transformations upon LMWH engagement in the biologically-active complexes three reference simulations were performed: of IFNγ and IL-6 apo forms (500 ns, resp. 150 ns), as well as of the complex IL-6/IL-6Rα (150 ns), as in this case, LMWH hinders also the formation of the triple complex with the gp130 receptor.

### 4.8. Complex-Interactions Analysis

The evaluation of the interactions in the investigated molecular complexes was performed with the MOE software suit. To this end, the MD-generated structures were further optimised in the MMFF94 force field to allow for a hydrophobicity profiling of the respective solvent accessible surface. The interactions between IL-6 or IL-6/IL6Rα and the heparin molecules were analysed and visualised with the ligand interactions functionality of MOE [83,84]. 

### 4.9. Cell Culture

WISH cell line (ATCC^®^ CCL-25™, ATCC, Manassas, VA, USA) was propagated in Eagle’s Minimum Essential Medium (EMEM, ATCC^®^ 30-2003™, ATCC, Manassas, VA, USA) supplemented with 10% fetal bovine serum (Gibco™, Waltham, MA, USA) and penicillin-streptomycin (10000 U/mL, Gibco™, Waltham, MA, USA). Cells were cultured in 25-cm^2^ flasks (Thermo Scientific™ Nunc™, Waltham, MA, USA) at 37 °C incubator with 5% CO_2_. 

### 4.10. Inhibitory Antiproliferative Assay 

Confluent WISH cell monolayers were detached by trypsin-EDTA treatment (Sigma-Aldrich, St. Louis, MO, USA) and were seeded in a 96-well plate (Corning^®^, Corning, NY, USA) at a density of 1 × 10^6^ cells/mL. Cells were treated with:

- 100 ng/mL IFNγ purified as described in [13],
- IFNγ (100 ng/mL) supplemented with Fraxiparine (GlaxoSmithKline Pharmaceuticals S.A.) in concentrations varying from 1.5 to 150 anti-Xa IU/mL pre-incubated for 1 h at room temperature, and
- Fraxiparine in the same concentrations as in ii. Further, the antiproliferative activity of IFNγ was measured by a modified kynurenine bioassay as described earlier [12].

### 4.11. Cellular Localisation of Phosphorylated STAT1

In order to determine the localisation of the phosphorylated STAT1, WISH cells were grown on coverslips, washed twice with 1 × PBS, and treated for 15 min with 100 ng/mL IFNγ alone or IFNγ (100 ng/mL) pre-incubated for 1 h at room temperature with Fraxiparine in concentrations varying from 7.5 to 150 anti-Xa IU/mL. As a control, the cells were separately treated with the highest concentration of Fraxiparine (150 anti-Xa IU/mL). Following the incubation, the cells were first fixed with 3.7% formaldehyde in 1 × PBS for 7 min at room temperature followed by washing with 1 × PBS. Next, the cells were fixed with methanol for 5 min at −20 °C. After the fixation, the cells were permeabilised with 0.5% Triton X-100 in PBS for 5 min, washed with 1 × PBS and blocked for 1 h at room temperature with blocking buffer (5% BSA, 0.1% Tween 20 in 1 × PBS). The cells were incubated overnight at 4 °C with rabbit anti-phospho-STAT1 (Tyr701) primary antibody (Cell Signaling Technology, 9167S, Danvers, MA, USA) diluted 1:100 in blocking buffer, followed by washing 5 times with 0.1% Tween 20 in 1 × PBS. Cells were then stained with secondary antibody Alexa Fluor^®^ 594 (Thermo Fisher Scientific, Waltham, MA, USA) goat anti-rabbit IgG in blocking buffer at dilution 1:500 for 1 h at room temperature in the dark. Following the incubation, the coverslips were washed 4 times with 0.1% Tween 20 in 1 × PBS. The nuclei were counterstained with 0.5 μg/mL DAPI (Sigma-Aldrich, St. Louis, MO, USA) for 2 min at room temperature. The coverslips were washed with distilled water and mounted on glass slides with ProLong™ Gold Antifade mounting media (Invitrogen, Waltham, MA, USA). Images were analysed by CellProfiler software [85].

## 5. Conclusions

There is a considerable volume of observational data regarding the use of low-molecular-weight heparin in the treatment of COVID-19 because of its anti-coagulatory effect. Our investigations reveal another facet of heparin: a potent anti-inflammatory agent due to its ability to engage with two of the key cytokines in the development of the cytokine storm, IFNγ and IL-6, thus downregulating their biological activity. This observation underpins the possibility for heparin to favourably influence conditions characterised by an overexpression of certain cytokines. Such conditions are associated not only with autoimmune diseases, but also with uncontrolled inflammatory processes, in particular with COVID-19, where they often lead to a lethal exit. We refer to extensive evidence from clinical observations of the positive outcome of heparin treatment in COVID-19 patients. Our findings suggest that this outcome may even be better if the administration of heparin (meaning LMWH) is started earlier, due to its anti-cytokine activity in addition to clotting prevention.

## Supplementary Materials

The following are available online at https://www.mdpi.com/article/10.3390/ijms221910730/s1, Figure S1. Pair contacts between IFNγ and the four hexasaccharides; Figure S2. Solvent accessible surface area (SASA) of the hexasaccharides; Figure S3a,b. Secondary structure plots of IFNγ: reference simulation and IFNγ in complex with four representative hexasaccharides (3 independent runs); Figure S4. C-termini solvent-accessible surface area (SASA); Figure S5. Hydrogen bonds formed between IFNγ and each of the hexasaccharides; Figure S6. Electrostatic potential surface of the starting structure of IFNγ (left panel) and of IFNγ in complex with the four sample hexasaccharides (right panel); Figure S7. The IL6/IL6Rα/gp130 complex; Figure S8. Secondary structure plots of IL-6: reference simulation (top panel); binding simulation with one LMWH molecule (bottom panel); Figure S9. Secondary structure plots of IL-6/IL-Rα complex: reference simulation (top panel); binding simulation with one LMWH molecule (bottom panel); Figure S10. The hexasaccharide used in the MD simulations as a representative LMWH molecule.
