# Supplementary material for: Molecular Mechanism of the Anti-Inflammatory Action of Heparin
Source: Int J Mol Sci. 2021 Oct 3;22(19):10730. doi: 10.3390/ijms221910730 (PMC8509397; doi:10.3390/ijms221910730)
Supplement: Supplementary file 1 [file ijms-22-10730-s001.zip › ijms-1384000-supplementary.pdf]

Supplementary Material

L. Litov et al., *Molecular mechanism of the anti-inflammatory action of heparin*

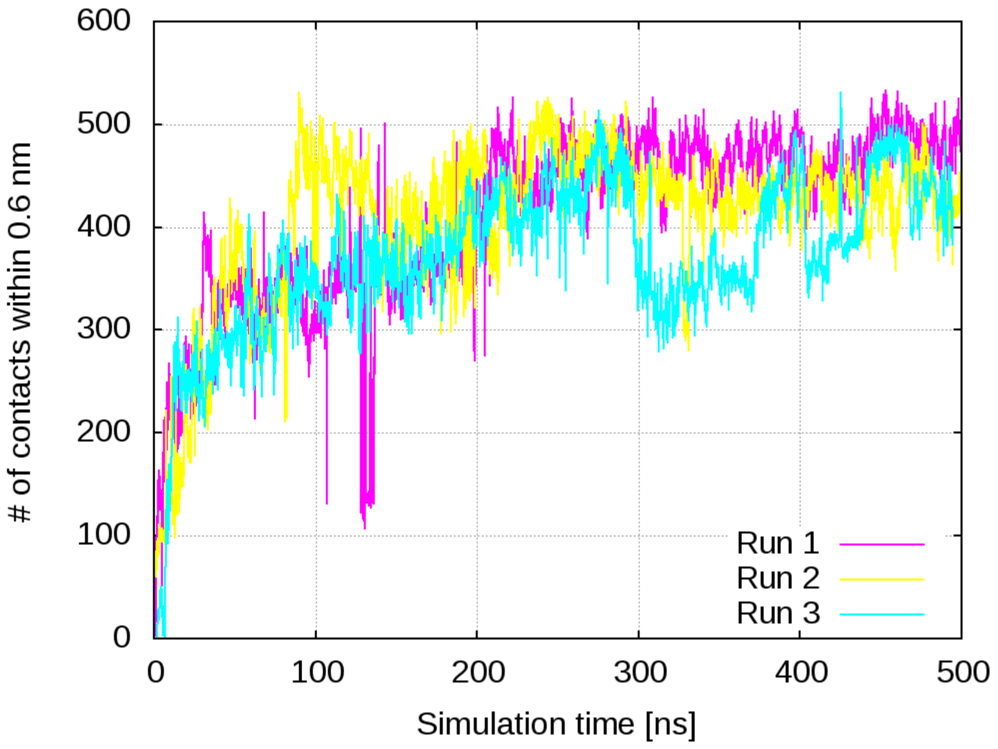

FIGURE S1. Pair contacts between IFNγ and the four hexasaccharides. Number of contacts as a function of the simulation time between any pair of atoms of IFNγ and any of the four hexasaccharides within 0·6 nm: run 1 (magenta), run 2 (yellow), and run 3 (cyan).

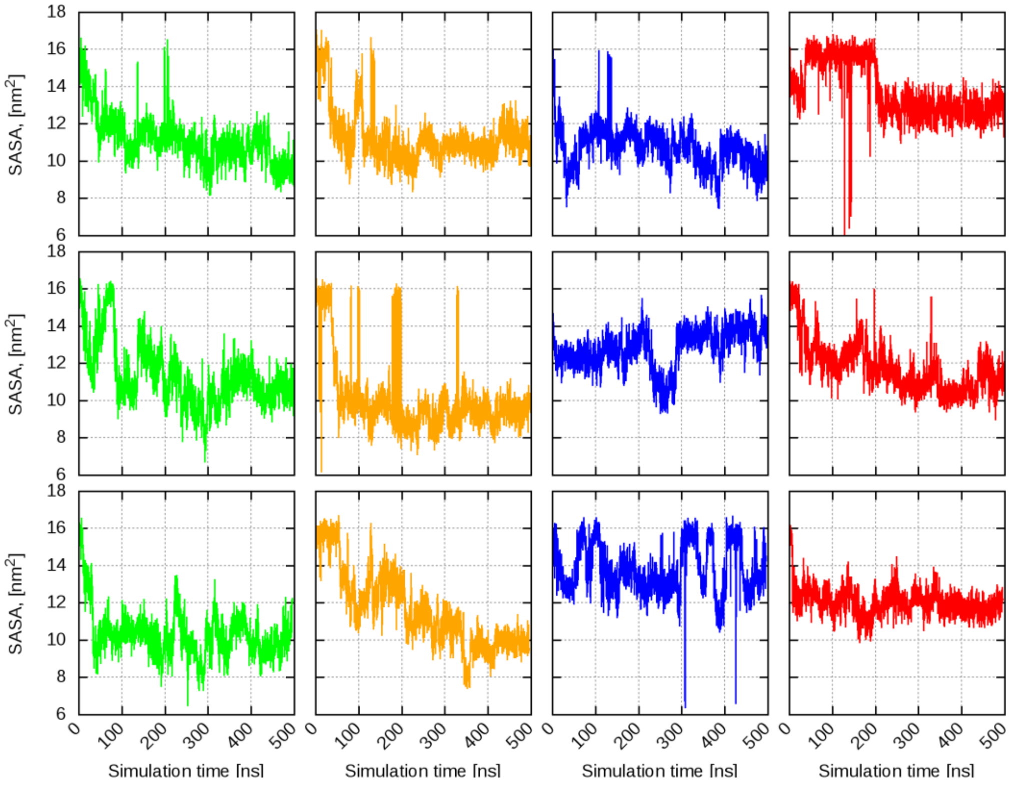

FIGURE S2. Solvent accessible surface area (SASA) of the hexasaccharides: dp6_1 – green, dp6_2 – orange, dp6_3 – blue, and dp6_4 – red, for run 1 (upper panel), run 2 (middle panel), and run 3 (bottom panel).

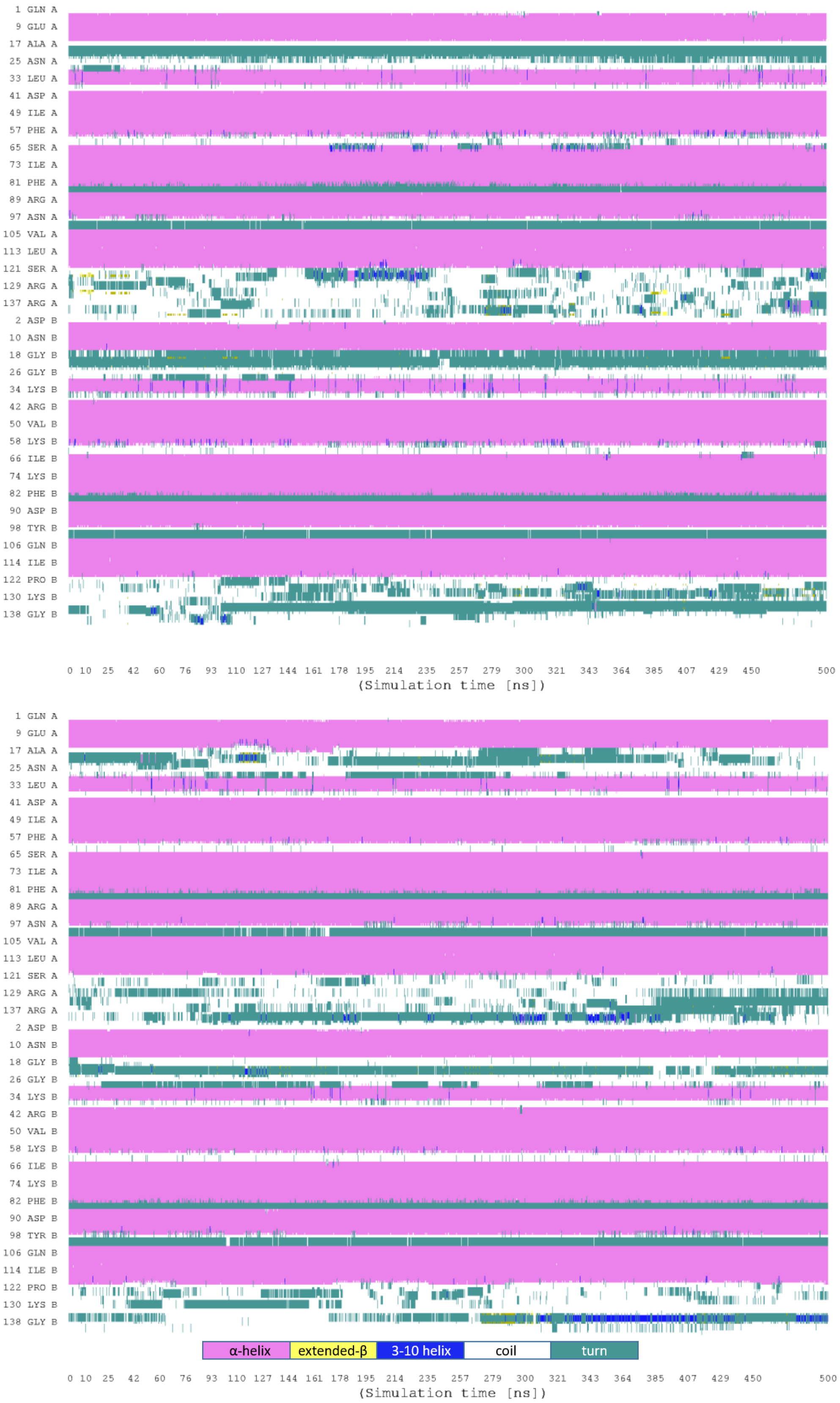

FIGURE S3a. Secondary structure plots of IFNγ: reference simulation (top panel); IFNγ in complex with four representative hexasaccharides (run 1, bottom panel).

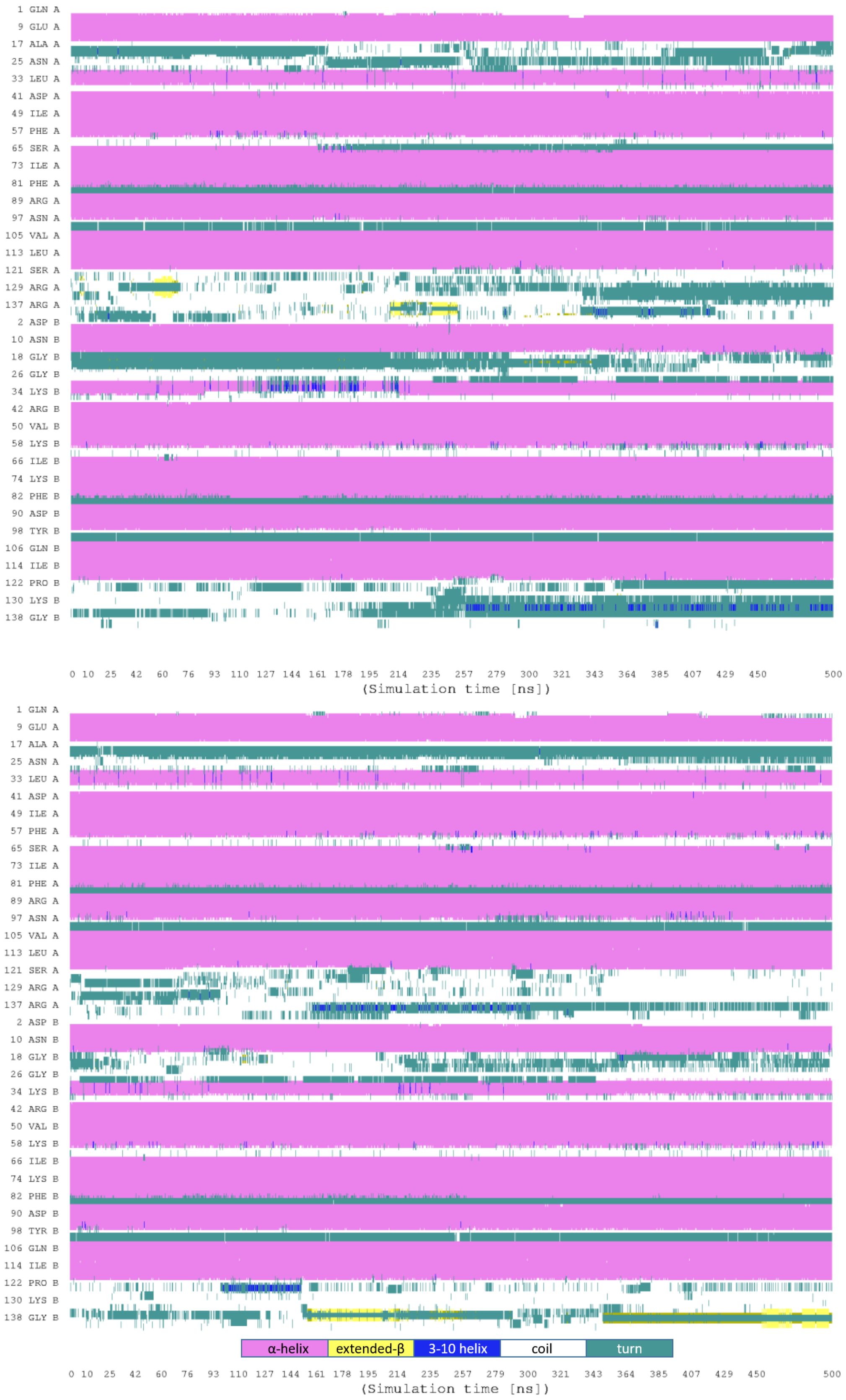

FIGURE S3b. Secondary structure plots of IFNγ: IFNγ in complex with four representative hexasaccharides – run 2 (top panel), and run 3 (bottom panel).

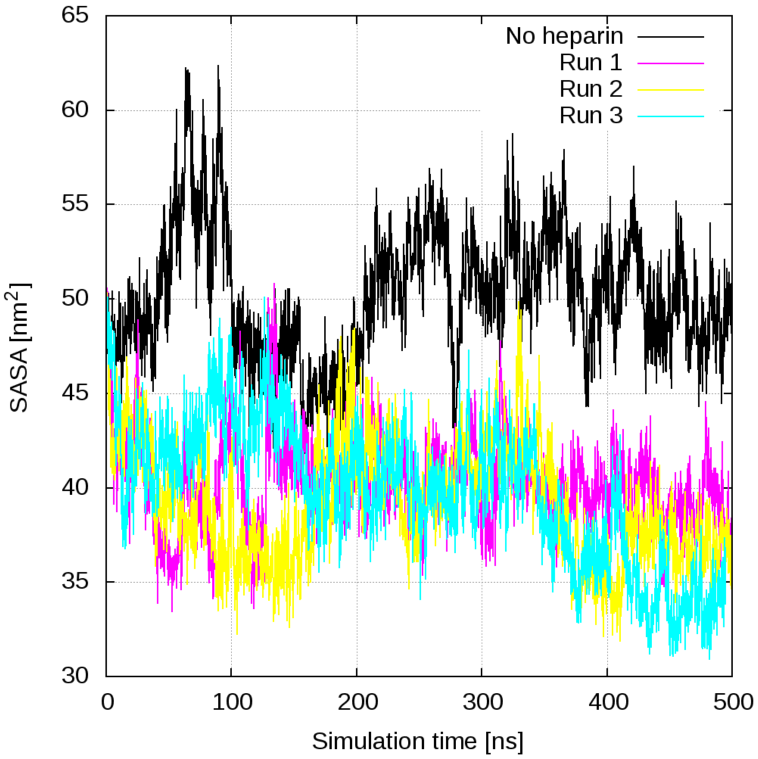

FIGURE S4. C-termini solvent-accessible surface area (SASA): IFNγ reference simulation (in black) and the three independent binding simulations, resp. in magenta, yellow, and cyan.

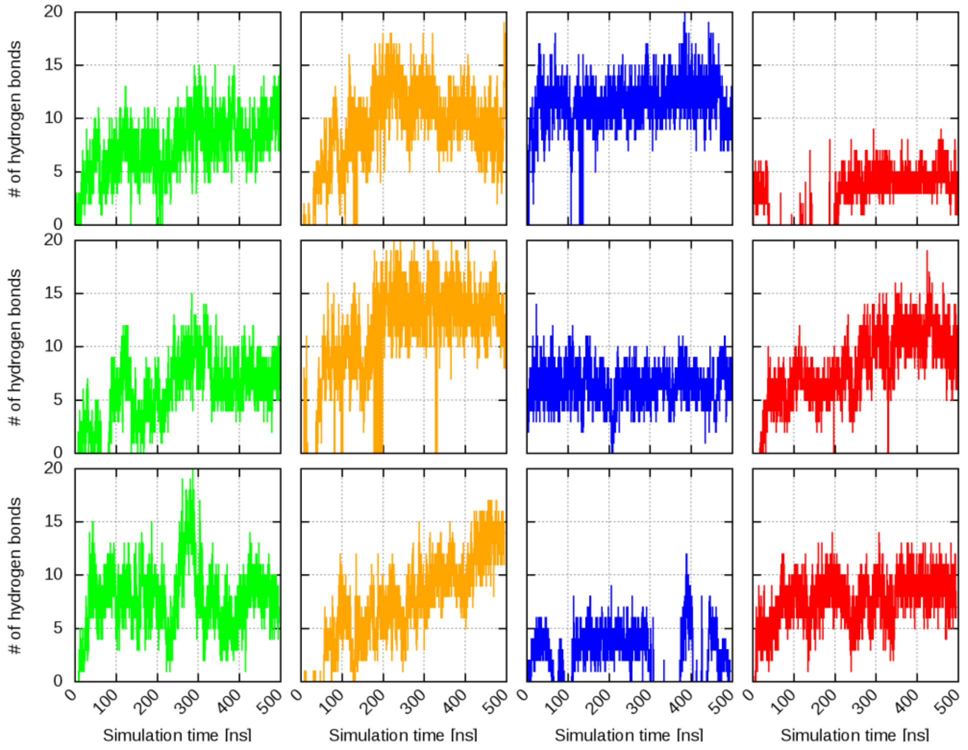

FIGURE S5. Hydrogen bonds formed between hIFNγ and each of the hexasaccharides: dp6_1 – green curve, dp6_2 – orange curve, dp6_3 – blue curve, and dp6_4 – red curve, for run 1 (upper panel), run 2 (middle panel), and run 3 (bottom panel).

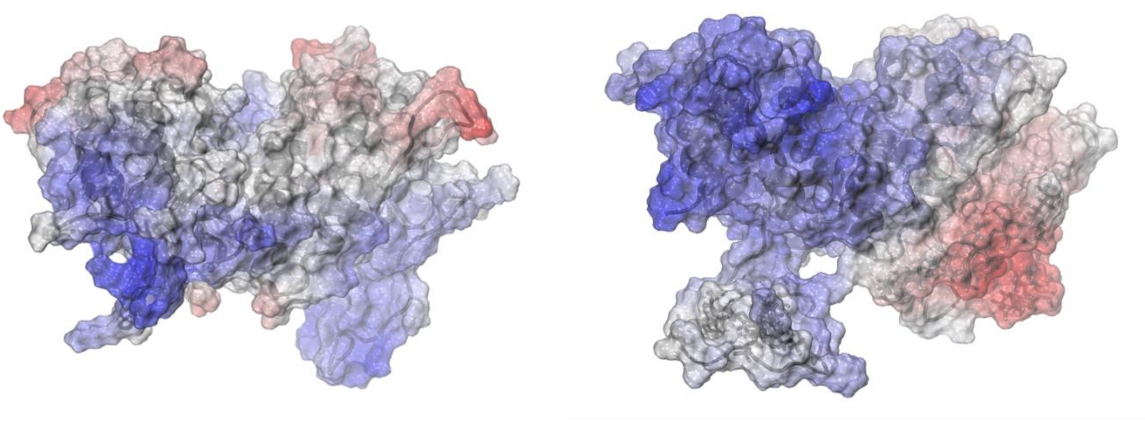

FIGURE S6. Electrostatic potential surface of the starting structure of hIFNγ (left panel) and of hIFNγ in complex with the four sample hexasaccharides (right panel). Positively charged areas are coloured in blue, negatively charged areas – in red.

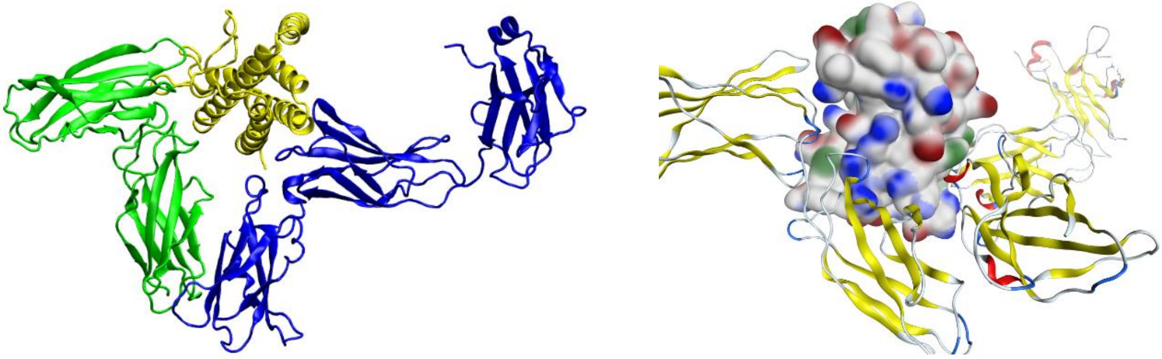

FIGURE S7. The IL6/IL6Rα/gp130 complex. (A) IL-6 in yellow, IL-6Rα in green, gp130 in blue; (B) IL-6 molecule presented with its SAS (hydrophobic regions in green, positively charged regions in blue, negatively charged regions in red), IL-6Rα and gp130 molecules (in ribbon representation) are to the left, resp. to the right of the IL-6 molecule.

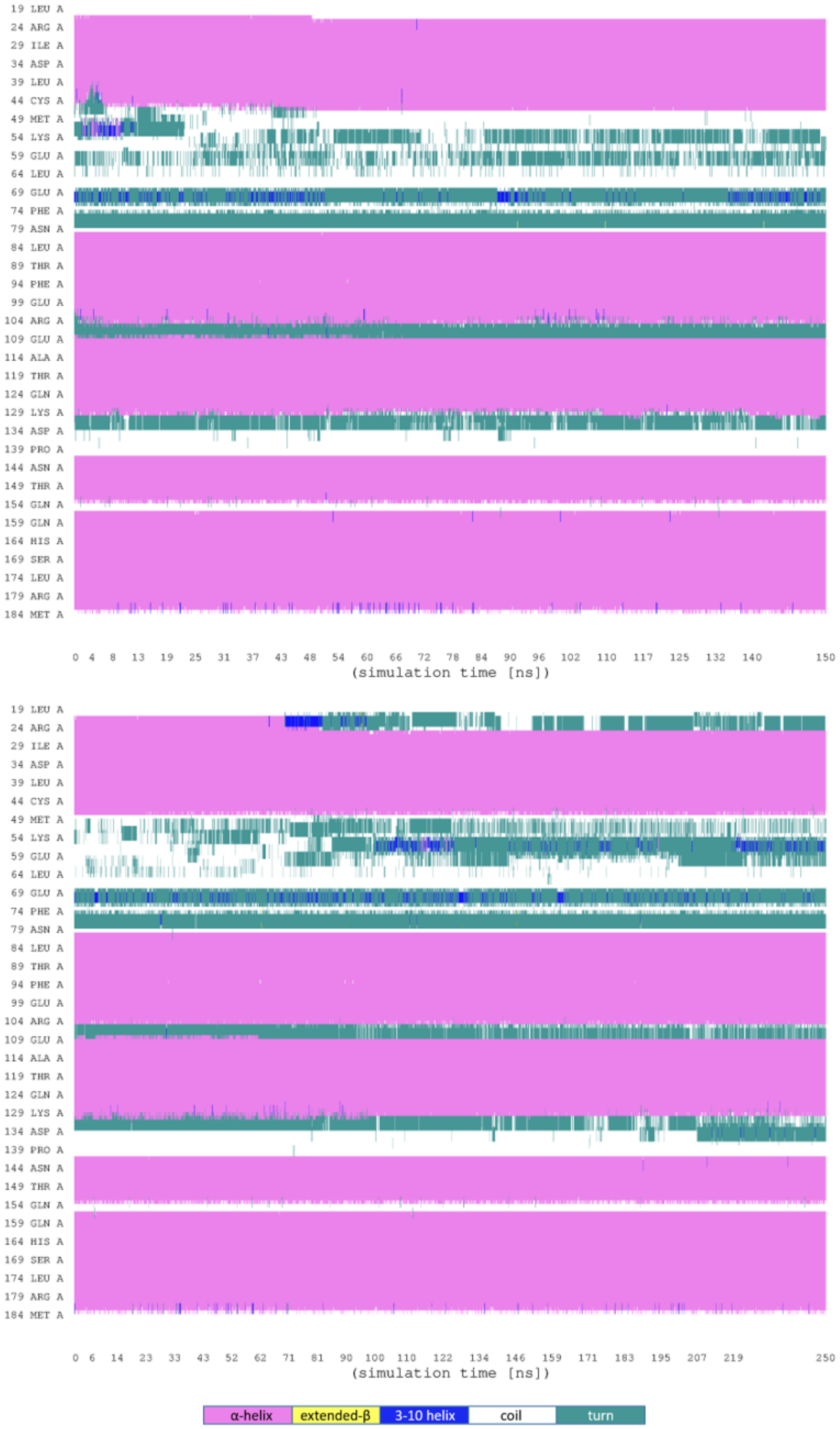

FIGURE S8. Secondary structure plots of IL-6: reference simulation (top panel); binding simulation with one LMWH molecule (bottom panel).

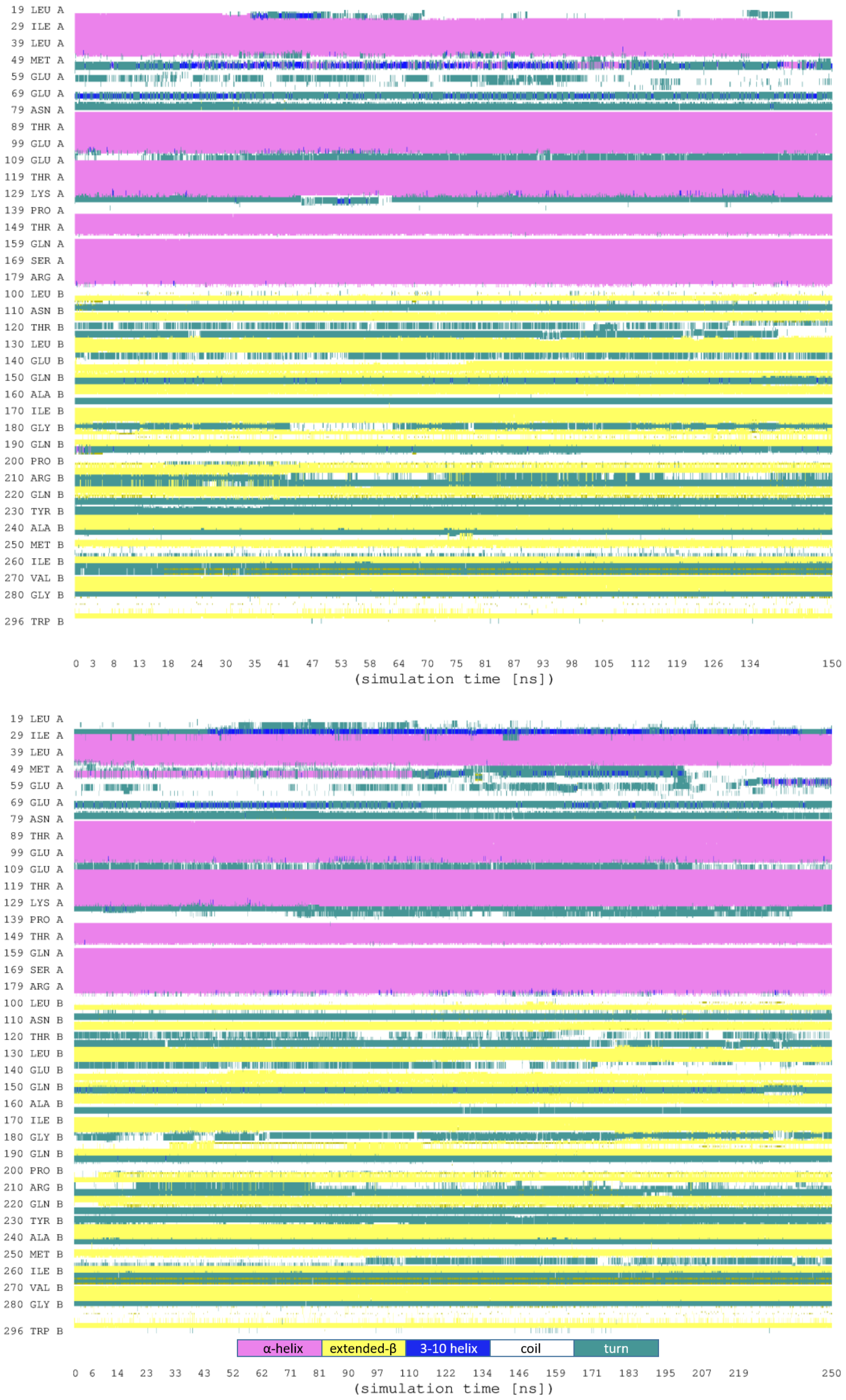

FIGURE S9. Secondary structure plots of IL-6/IL-Rα complex: reference simulation (top panel); binding simulation with one LMWH molecule (bottom panel).

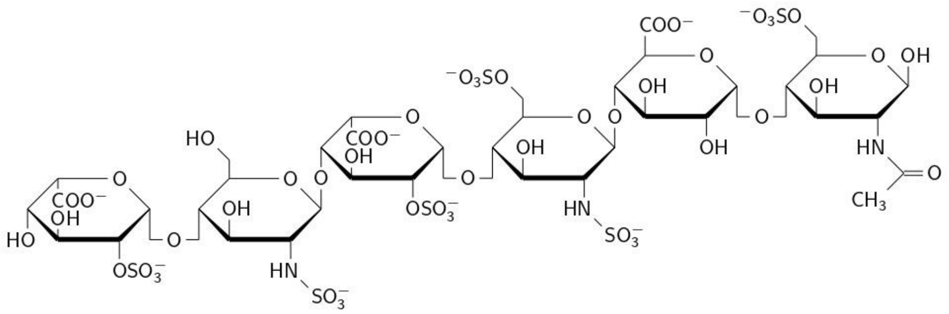

FIGURE S10. The hexasaccharide used in the MD simulations as a representative LMWH molecule: α-L-IdoA(2S) (1→4) β-D-GlcNS (1→4) α-L-IdoA(2S) (1→4) β-D-GlcNS(6S) (1→4) β-D-GlcA(1→4) β-D-GlcNAc(6s).
